# Factors influencing user’s health information discernment abilities in online health communities: based on SEM and fsQCA

**DOI:** 10.3389/fpubh.2024.1379094

**Published:** 2024-09-16

**Authors:** CaiPing Wei, Yufeng Cai, Jianwei Liu, Yi Guo, Xusheng Wu, Xiaofeng He, DeHua Hu

**Affiliations:** ^1^Department of Biomedical Informatics, School of Life Sciences, Central South University, Changsha, China; ^2^Shenzhen Health Development Research and Data Management Center, Shenzhen, China

**Keywords:** online health communities, health information discernment abilities, information ecology theory, perceived value, fsQCA

## Abstract

**Introduction:**

Online health communities have become the main source for people to obtain health information. However, the existence of poor-quality health information, misinformation, and rumors in online health communities increases the challenges in governing information quality. It not only affects users’ health decisions but also undermines social stability. It is of great significance to explore the factors that affect users’ ability to discern information in online health communities.

**Methods:**

This study integrated the Stimulus-Organism-Response Theory, Information Ecology Theory and the Mindsponge Theory to constructed a model of factors influencing users’ health information discernment abilities in online health communities. A questionnaire was designed based on the variables in the model, and data was collected. Utilizing Structural Equation Modeling (SEM) in conjunction with fuzzy-set Qualitative Comparative Analysis (fsQCA), the study analyzed the complex causal relationships among stimulus factors, user perception, and the health information discernment abilities.

**Results:**

The results revealed that the dimensions of information, information environment, information technology, and information people all positively influenced health information discernment abilities. Four distinct configurations were identified as triggers for users’ health information discernment abilities. The core conditions included information source, informational support, technological security, technological facilitation, and perceived risk. It was also observed that information quality and emotional support can act as substitutes for one another, as can informational support and emotional support.

**Discussion:**

This study provides a new perspective to study the influencing factors of health information discernment abilities of online health community users. It can provide experiences and references for online health community information services, information resource construction and the development of users’ health information discernment abilities.

## 1 Introduction

With the rapid advancement of artificial intelligence and Internet technology, various online health services have emerged one after another, offering opportunities to transform the traditional healthcare industry (1–3). The public’s health awareness and health literacy are also constantly improving, leading to an increasing demand for health information (4, 5). Online Health Communities (OHCs) serve as open online interactive platforms where users, including the general public, patients and their families, caregivers, and medical professionals, can engage in discussions about health and medical issues, seek expert consultations, share treatment experiences, and seek social support (6, 7). OHCs play a crucial role in disseminating health information, assisting users in making health Decisions, and preventing the occurrence of diseases. As a new model of “Internet + medical health,” OHCs have rapidly emerged and developed (8). Additionally, OHCs can help reallocate idle medical resources, promote more efficient use of medical resources, solve the problem of mismatch between supply and demand of health services, and thereby improve the doctor-patient relationship (9–11). According to the 53nd Statistical Report on China’s Internet Development released by the China Internet Network Information Center (CNNIC), as of December 2023, the number of Chinese Internet users has reached 1.092 billion, with an internet penetration rate of 77.5%. The number of Chinese Internet healthcare users has reached 414 million, accounting for 37.9% of the overall Internet user base (12). Internationally renowned Internet healthcare platforms such as Haodaifu, Doctor on Demand, and Patients Like Me have risen rapidly around the world (13, 14). Online health communities have become a vital source for people to obtain health information and occupy an important position in the modern healthcare industry.

Due to the openness, commerciality, and profit-seeking nature of online platforms, information quality problems emerge one after another in OHCs, such as the spread of false and difficult-to-distinguish health information to gain users attention and information epidemics, which have increased the difficulties of information quality governance in these communities (15, 16). Exposure to distorted information inevitably affects the future acceptance of accurate health knowledge. If they make judgments based on this invalid, incomplete, or even wrong information, it will not only affect people’s health Decisions, but also cause damage to social stability. For example, during the COVID-19 epidemic, the Iranian people blindly believed in unproven treatment methods. To prevent COVID-19, they consumed high-concentration alcohol, causing more than 600 deaths and more than 3,000 people being poisoned (17, 18). This posed a serious threat to public health, with harmful consequences for global health and wellbeing (19).

In recent years, countries around the world have taken various measures to improve the quality of information on online health platforms. For instance, the European Union has issued the Strengthened Code of Practice on Disinformation (20). China has released the Guidelines on the Establishment of a Sound Mechanism for the Publication and Dissemination of Health Science Knowledge Across the Media and established the China Internet Joint Rumor Refutation Platform (21). In addition, the Swiss Health On the Net Foundation has formulated the HONcode guidelines. From the perspective of information technology, scholars have explored false information filtering technology and health information monitoring technology. Guo and Wei (22) used block matching and fuzzy neural networks to effectively improve the classification and identification efficiency of false information on Weibo, specifically about China’s context. Wan et al. (23) established a database of false drug advertisements and real drug advertisements on Sina Weibo, and confirmed that the use of a support vector machine classifier to classify false drug advertisements has the best effect, providing an effective method for the government to identify and combat false advertisements.

Scholars have conducted relevant studies on health information discernment from multiple perspectives. Wang and Zhou (24) explored the current status of the health information discernment abilities of the older adult in the social media environment, and evaluated the impact of learning a pseudo-health information feature list on improving their discernment abilities. Zhang (25) explored the status of college students’ health information discernment abilities in a complex online environment and proposed specific strategies to enhance their health information discernment abilities. According to Li and Zhang (26), college students’ health information discernment abilities were mainly affected by their location of birth, family income, and attention to health information. Hou and Yang (27) used semi-structured interviews to collect data on the health information discernment ability of urban residents in China, and constructed a model of factors influencing health information discernment ability. These factors mainly include personal characteristic factors, information factors, institutional factors, and social class factors. Qiu et al. (28) identified that the health information discernment abilities of WeChat users were most significantly influenced by content updates, information readability, and government propaganda.

In terms of research perspectives, most studies focus on various demographic groups of internet users and social media users. Eysenbach et al. (29) found that factors such as the authority of information sources, website layout and appearance, readability of information, and authoritative certifications significantly impact the health information discernment ability, through interviews with internet users. Zhang and Li (30) conducted a survey among internet users of different age groups and discovered that age, gender, place of residence, and the level of interest in health information significantly influence the health information discernment abilities. However, most studies predominantly take “internet users” and “social media users” as a whole research subject, neglecting to specifically focus on users of online health communities (OHCs), who typically have a stronger need for health information. OHCs users are generally more actively engaged with health information compared to regular internet users. Unlike other social media platforms, OHCs are specialized in providing online health services, making them more vertical and targeted. Users participate in these communities to improve their health status, sharing common health goals. They actively discuss health issues, share experiences, and seek social support or informational support related to their specific health concerns. This interaction among users fosters a sense of resonance and cohesion. Health information disseminated among users through homogeneous communication significantly increases its credibility and propagation power. However, the large amount of indistinguishable true and false health information in OHCs poses significant risks. If false or incorrect information is trusted and widely spread, it can adversely affect users’ health Decisions, making it critical to enhance the ability of OHC users to discern health information.

In terms of research methods, some studies utilized computer technology to explore health information detection (31, 32). While more studies relied on qualitative interviews and grounded theory to examine factors influencing individual discernment abilities (27, 33, 34). These methods possess a degree of subjectivity and May overlook the interrelationships among influencing factors (28). More objective methods are required to evaluate the importance of the influencing factors. Most of the studies focused on the net effect of a single influencing factor on the user’s health information discernment abilities, ignoring the synergistic effect of the configuration of multiple factors on the results. Nevertheless, users’ health information discernment activity is a complex Decision-making process. The process is often not the result of a single factor, and the intricate interrelationships among these factors remain to be explored.

To address this research gap, this study took OHCs users in China as the research object, utilizing Stimulus-Organism-Response Theory, Information Ecology Theory and the Mindsponge Theory as theoretical guidance. It comprehensively considered four factors: information, information environment, information technology, and information people. It makes up for the shortcomings of the study of a single factor, and aims to establish a comprehensive model of factors influencing users’ health information discernment abilities. In addition, this study combined Structural Equation Modeling (SEM) with fuzzy-set Qualitative Comparative Analysis (fsQCA), which avoids the limitation that SEM can only examine the net effect of a single variable that affects users’ health information discernment abilities. This combination allows for a more detailed and in-depth analysis and understanding of the combined influence of multiple factors. The configuration results provide suggestions for improving users’ health information discernment abilities and improving OHCs functions.

## 2 Research hypothesis and model construction

### 2.1 Theoretical background

#### 2.1.1 Information ecology theory

The concept of information ecology was first proposed by Horton (35). Information ecology theory views information, information people, information technology, and the information environment as a whole. It emphasizes the balance and development of humans, environment, and technology in the information ecosystem, so as to realize the production, dissemination, and utilization of information (36). Within this theoretical framework, the information people is the subject, controlling the information activities within the system. The information itself is the object, existing independently of human will. Information technology serves as the medium for information transmission. The information environment is the field where the interaction between the subject and object occurs. The core issue of this theory is the information behavior generated by the information people and the interactions between the information people and other elements (37).

As a classic theory in the field of explaining user information behavior, information ecology theory has been widely applied in studies on influencing factors of OHCs users’ information seeking behavior (38), information adoption behavior (39), information interaction behavior (40), and information sharing behavior (41). Ji and Li (40) explored the willingness for emotional interaction among OHCs users from an information ecology perspective, in which the information dimension includes topic relevance and topic value, the information environment dimension includes group connection and group identity, the information technology dimension includes perceived platform usefulness and perceived function ease of use, and the information people dimension includes health status and health experience. Gao et al. (42) applied the information ecology theory to study the information processing of online health community users, in which the information dimension includes information quality, the information environment dimension includes emotional support and privacy issues, the information technology dimension includes system quality, and the information people dimension includes self-efficacy. Zhang et al. (36) explored the behavior of seeking medical information assistance from an information ecology perspective among OHCs users, where the information dimension includes information accuracy, information relevance, and information timeliness; the information environment dimension includes social acceptability and platform trust; the information technology dimension includes perceived ease of use and perceived usefulness; and the information people dimension includes self-efficacy, health information literacy, and assistance-seeking experience.

Users’ discernment of health information is an important part of online health community activities, affecting users’ health Decisions and usage experience. The complex influencing factors in health information discernment can also be explained systematically and holistically from the perspective of information ecology theory (43). In this study, from the viewpoint of information factors, users not only need to examine the quality of health information, but also consider whether the source of health information is credible and authoritative, which provides good information resources for their own health Decisions. From the viewpoint of information environment, rich information support and emotional support realms help to create an efficient, orderly, and sustainable user interaction environment, thus improving the efficiency of obtaining correct health information and promoting the quality of community information services. From the viewpoint of information technology, information technology connects users with the community environment. The safe, intelligent, and convenient technology can expedite the realization of platform functions and provide a basic guarantee for the good experience of users. From the viewpoint of information people, users are the most active elements in the process of information active. The driving force and the confidence in the discerning task are related to the flow of health information in the community. Therefore, this study explores the factors influencing the health information discernment abilities of online health community users from four aspects: information (information quality, information source), information environment (informational support, emotional support), information technology (technological security, technological facilitation), and information people (self-efficacy).

#### 2.1.2 Stimulus-organism-response theory

Mehrablan and Russell (44) proposed that the general human behavior model is the “stimulus-organism-response” model, which is used to explain and predict the impact of stimuli from different environments on individual cognition, individual behavior, and personal emotions. The model consists of three parts: stimulus, organism, and response. Stimulus is defined as some kind of object, event, or characteristic, which can be divided into external stimulus and internal stimulus. Organism, as an intermediary variable, is an individual’s psychological transformation mechanism, such as emotional or cognitive changes, which indicates that the behavior of the organism will be affected by the influence of internal factors. Response represents the individual outcome variable, which generally manifests as an approach or avoidance of things (45).

This model has been widely used in studies on individual offline and online behaviors, such as information participation behavior (46). Recently, it has been used to study the online behaviors of individuals in the face of online disinformation and rumors (47). Based on Stimulus-Organism-Response theory, Liu et al. (48) took rumor type and rumor ambiguity as stimulus and perceived authenticity, perceived importance and perceived trust as the organism’s perceptual states, and verified the influence mechanism of individual’s intention to verify rumor. Wu (49) considered informational dependency and social dependency as stimulus, and investigated how such stimulus led to positive and negative effects within organisms, which in turn affects the individual’s perception of misinformation sharing. Xie and Hu (50) regarded perceived credibility as an external stimulus, subjective norms, individual norms, and attitudes as organic states, and the rumor deletion intention and the rumor disproving sharing intention as a response, verifying the rumor correction behavior of social media users and its influence paths.

Additionally, users’ perception of health information services in OHCs is closely related to their ability to discern health information. Perceived value is the overall utility assessment of health information attributes by users (51). From the perspective of perceived value, when users perceive that the health information they obtain can bring value to themselves, they generate positive emotions, adopt the information, and further enhance their ability to discern information. Perceived risk is the subjective judgment by users of potential negative outcomes perceived in online health information services (52). Perceived risks include false health information, privacy concerns, privacy breaches, and financial security, among others, which are crucial factors influencing users’ use of online health information services (53). Based on the S-O-R theory, Cui (54) found that users’ perceived risks have a negative impact on the dissemination of false information on the internet. For health information with higher perceived risks, users need to invest more effort in systematic and rational thinking. In this study, perceived value and perceived risk represent the users’ organism perception states, which affect their health information discernment abilities.

It can be seen that the S-O-R theory is very suitable for research on health information in online health communities. Based on the S-O-R theory theoretical framework, this study examined the health information discernment abilities of OHCs users. In this study, when users exchange information in an online health community, they are not only stimulated by external factors, including the information itself, the environment of the community, and information technology; they are also stimulated by their own factors, such as self-efficacy. Therefore, the “stimulus” refers to the information factor (information quality, information source), information environment (informational support, emotional support), information technology (technological security, technological facilitation), and information people (self-efficacy). Under the double stimulation of internal and external factors, the psychological perception of the user will change. This “organism” perceptual state changes, mainly expressed in the size of the perceived risk and perceived value judgment. Finally, the user’s “reaction,” that is, health information screening ability.

#### 2.1.3 Mindsponge theory

Vuong and Napier (55) proposed a new information processing mechanism: Mindsponge Theory. It describes the psychological mechanism of which a person accepts or rejects new external information (56). First, an individual receives external information through books, media, the Internet, etc. Then, the individual will sift and filter the information according to established values, beliefs, and cognitive frameworks, and categorize the information into useful and useless, credible and non-credible categories. Later, the information that is considered useful and credible will be absorbed and integrated into the individual’s cognitive structure, which is similar to the process of a sponge sucking up water, and rejecting incompatible information. The individual May adapt and reconfigure the existing cognitive structure to fit the new information and environment. Ultimately, the reconfigured cognitive structure influences the individual’s feedback to the outside world.

This theory has been used to study vaccine production (57), suicidal behavior (58), and health information processing (57, 59). For example, Tanemura et al. (60) explored the differences in individuals’ trust levels of risk-only negative health information and benefit–risk negative health information based on the Mindsponge Theory. They found that individuals’ trust levels of risk-only negative health information were higher than those of benefit–risk negative health information. Mindsponge theory was also applicable in explaining that learning interventions for individuals that improve individuals’ ability in recognizing unreliable COVID-19 information (61).

In this study, when a user receives new health information from an online health community, the user goes through the brain’s filtering system to weigh its perceived value and perceived risk. When an individual’s perceived value of health information is greater than the risk, then the resulting high level of trust promotes health information uptake. Conversely, when risk outweighs value, i.e., when the perceived adverse consequences of new health information for an individual’s health Decisions outweigh its convenience, and health information is perceived to be unreliable, then health information is rejected. In conclusion, users’ health information screening activity is an uninterrupted and continuous process of selective information absorption and exclusion in order to maintain psychological and cognitive balance in order to adapt to the complex online health community information environment, thus realizing the improvement of health information discernment abilities.

### 2.2 Model construction

**Figure 1:**
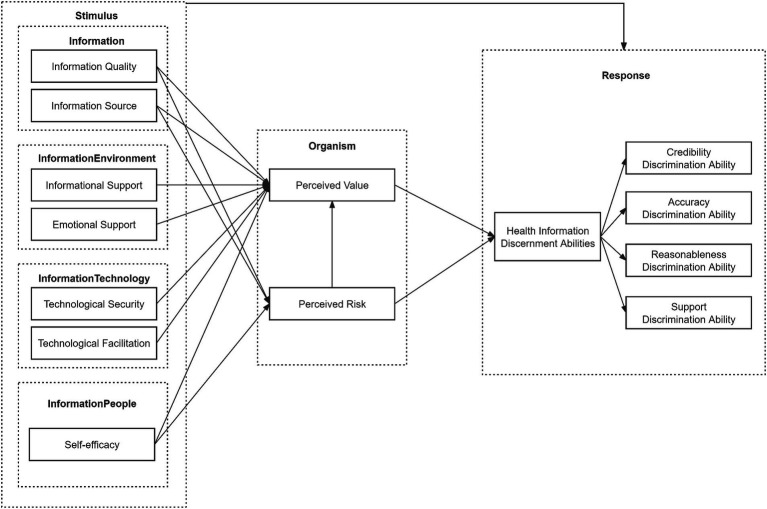
Structural research model.

Based on the information ecology theory, the stimulus-organism-response theory and the Mindsponge theory, this study developed a research model with four dimensions influencing factors: information (information quality, information source), information environment (informational support, emotional support), information technology (technological security, technological facilitation) and information people (self-efficacy). These dimensions serve as the stimulus factors in the research model. Perceived value and perceived risk, two factors that can reflect the user’s psychological perception, are used as the perceptual state in the organism. The user’s health information discernment abilities are regarded as the individual’s response, which mainly composed of credibility discrimination ability, accuracy discrimination ability, reasonableness discrimination ability and support discrimination ability. Therefore, the research model constructed in this study is shown in Figure 1.

### 2.3 Research hypothesis

#### 2.3.1 Information, user perception, and health information discernment abilities

In this study, information elements mainly included information quality and information sources. Information quality is a measure of the accuracy, truthfulness, and completeness of health information (62). The health information searched in OHCs includes advice and information on physical conditions, symptoms, and treatment options, which mainly guides users’ health Decisions. Obviously, the accuracy and authenticity of information are crucial. When persuasive and high-quality health information is searched for, it can enhance users’ perceived benefits from the information, thereby promoting perceived value and better satisfying their own information needs (63). On the contrary, the reliability of information is considered to reduce an individual’s perception of risk. Shah and Wei (64) conducted a survey on 630 users during the COVID-19 pandemic and found that source credibility and information quality are significantly positively correlated with perceived value and significantly negatively correlated with perceived risk. Sun and Wu (65) showed that the comprehensiveness and accuracy of COVID-19 information content can affect the public’s perceived risk. When health information is incomplete or lacks logical consistency, it can mislead users, increase their concerns, and elevate their psychological risk levels. Therefore, individuals with high perceived risk require more convincing arguments, such as higher quality health information, to reduce their risk perception. Liang et al. (66) drawing on rational choice theory, studied the online health information use of physically disabled individuals and found that information quality and system quality can increase perceived benefits and reduce perceived risks. Therefore, three hypotheses are proposed as follows:

H1a: Information quality positively influences perceived value.

H1b: Information quality negatively influences perceived risk.

H1c: Information quality positively influences health information discernment abilities.

The information source considers the credibility of the information source, independent of the information’s content. In OHCs, users can act as both creators and disseminators of health information, and their knowledge and experience vary. Hence, the source of information is an important factor in information behavior research (67). Shareef et al. (68) pointed out that when the source credibility is low, the recipients discount the value of the information content they receive. Information from authoritative and professional sources generally has a low perceived risk to users, while information from user-generated content and commercial sources May increase the perceived risk to users. Cha et al. (69) showed that users are more likely to have positive cognitive and affective responses to information obtained from Weibo that have a high level of credibility in its sources. Thompson (70) points out that perceived risk is negatively correlated with the trust of the Internet as an information source. This means that as people’s trust in these sources of information Decreases, their perception of risk to health information increases. At the same time, clear information sources are more conducive for users to judge the authenticity of health information. Therefore, three hypotheses are proposed as follows:

H2a: Information source positively influences perceived value.

H2b: Information source negatively influences perceived risk.

H2c: Information source positively influences health information discernment abilities.

#### 2.3.2 Information environment, user perception, and health information discernment abilities

In this study, the elements of the information environment mainly included informational support and emotional support. Users engage in online discussions and interactions on the internet to obtain specific social support, including emotional, informational, companionship, and technical support (71). Informational support means that users obtain health and medical-related information through searching, consulting, answering, and discussing, including information on medications, healthcare measures, and treatment suggestions (72). Thus, OHCs facilitate the acquisition and sharing of health information and knowledge. According to the Mindsponge theory, when members of OHCs provide valuable advice or timely help to each other, users identify with these new values and assimilate and transform the health information into health knowledge, which is conducive to users’ reduction of informational uncertainty and making the right health Decisions, and facilitates their future self-health management. Hu et al. (73) used a case analysis method to conduct an in-depth analysis of the formation mechanism of patients’ perceived value, and found that sharing information among members is the key to improving patients’ perceived value. Therefore, the following hypotheses are made:

H3a: Informational support positively influences perceived value.

H3b: Informational support positively influences health information discernment abilities.

Emotional support is expressed as the respect, acceptance, or emotional comfort and encouragement received when an individual is in pain (74). Adequate emotional support is a key factor in creating a favorable atmosphere of interaction that promotes the sustainability of an online health community. Park et al. (75) stated that empathy and encouraging messages in OHCs can provide both emotional support and informational support to members. Emotional support can provide positive energy to other members, making community members feel more confident, in control, and empowered in order to address similar health issues. Receiving emotional support is also an important source of perceived value for users. Kanthawala and Peng (76) found a positive correlation between perceived emotional support and perceived message credibility. In addition, Sun (77) believes that emotional support, as a social resource, is a significant factor affecting the older adult’s health information discernment abilities. Therefore, the following hypotheses are made;

H4a: Emotional support positively influences perceived value.

H4b: Emotional support positively influences health information discernment abilities.

#### 2.3.3 Information technology, user perception, and health information discernment abilities

In this study, information technology elements mainly included technological security and technological facilitation. Technological security in OHCs refers to the security of hardware, software, and related data in online health communities. In the era of big data, the value of information and data is increasing day by day. The subsequent leakage of user privacy information or data loss has become more and more serious. Users have increased concerns about the security of information technology. Studies have shown that in OHCs, the higher the technological security, the more willing users are to share information (52). Internet users can help form a sense of belonging to websites with higher security, thereby further increasing perceived value (78). To enhance security levels from a technological standpoint, it is important to implement advanced data encryption measures, while deploying multi-layered access control mechanisms to ensure that only authorized users can access sensitive information, thereby reducing the risk of information misuse or unauthorized access (79). Additionally, establishing a scientifically efficient information review mechanism is essential to ensure the reliability of information sources, reducing the spread of false and misleading content, and thereby improving the overall quality of information. In such an environment, users can more quickly identify and filter out reliable health information, continuously learn, absorb, and increase their knowledge; ultimately enhancing their discernment capabilities. Some scholars believed that relevant departments must strengthen supervision of the quality of online health information to ensure the security of platform information, thus strengthening the ability of users to resist the interference of misinformation (80). Therefore, the following hypotheses are proposed:

H5a: Technological security positively influences perceived value.

H5b: Technological security positively influences health information discernment abilities.

The page layout, response speed, functional diversity, and convenience of OHCs all affect users’ usability experience and the ease of distinguishing health information. Technological facilitation emphasizes the promotion and support of information technology on people’s information behavior. For example, when an OHC has a user-friendly page layout and provides a clear and concise operation guide, it saves users’ learning cost and reduces difficulty in using it. Ease of use has a positive impact on users’ willingness to use a platform (81). Scholars have found that the difficult interoperability of the platform is one of the barriers to the public’s use of online platforms to obtain useful information (82). When constructing online health community information services, leveraging big data technology to deeply analyze users’ website browsing records, search habits, clicking patterns, and browsing durations can accurately capture users’ interests and health needs (83).This allows for intelligent and personalized health information recommendations, sparing users the trouble of sifting through vast amounts of information to find what they need. Additionally, providing users with other health information verification tools, such as recommending authoritative health information websites or fake health information features lists, to help users acquire, screen and validate health information more efficiently. Tang et al. (84) found that during the COVID-19 pandemic, the subjective and objective technical support conditions positively influenced the adoption behavior of government short videos. Improving OHCs functions and enriching user experience through technological means improves users’ understanding and judgment of health information in several ways. This enables users to more accurately discern valuable health information. Therefore, the following hypotheses are proposed:

H6a: Technological facilitation positively influences perceived value.

H6b: Technological facilitation positively influences health information discernment abilities.

#### 2.3.4 Information people, user perception, and health information discernment abilities

The information people element in this study mainly consisted of self-efficacy. Warner and Schwarzer (85) argued that general self-efficacy is an overarching self-efficacy of users when facing various challenges or confronting new things. This self-efficacy competence will largely influence people’s presumptions about the ability to implement or organize activities. Stronger self-efficacy can better withstand the negative impact of uncertainty factors in the network environment, the easier it is to form a better quality perception and value recognition of the results, resulting in lower perceived risk (86). Wang et al. (87) suggested that the self-efficacy of older adult users who have been in different contexts of family, community, and education for a long time affects their health information needs. Older adult users with high self-efficacy were more adept at expressing differences in the efficacy of health information products, and had a certain degree of ability to discriminate health information products. Therefore, the following hypotheses are made:

H7a: Self-efficacy positively influences perceived value.

H7b: Self-efficacy negatively influences perceived risk.

H7c: Self-efficacy positively influences health information discernment abilities.

#### 2.3.5 User perception and health information discernment abilities

User perception elements mainly included perceived risk and perceived value. Users assess the utility of health information when using OHCs, retaining information that is valuable for their health needs and refusing to access information that does not deal with their health risks (88). In this study, the perceived usefulness of health information, the emotional pleasure, and the establishment of good interpersonal relationships, as perceived by users, are all manifestations of perceived value. The perceived risk of online health information refers to the user’s perception and assessment of the risk level conveyed by the health information, and the subjective judgment and perception of the degree of threat to personal privacy information, physical health, or personal property. According to the Mindsponge theory point of view, the user’s processing of health information can be understood as a comparison of the perceived value and perceived risk of new information based on their own existing health knowledge and health experience. The higher the perceived value and the lower the perceived risk of health information, the more likely it is for the user to acquire and utilize. Liu et al. (89) used the S-O-R theory as a theoretical framework and constructed a model of the impact of mobile Medical App content presentation on user adoption willingness influence model. The results show that perceived value plays a mediating role, and the user’s perceived value of mobile medical APP content positively influences user adoption behavior. This indicates that health information with a higher value is more likely to capture users’ attention, stimulate their interest in in-depth exploration and learning, and motivate them to analyze and validate the information more carefully to enhance their trust in it. This process enhances users’ overall health literacy, thereby improving their ability to discern health information.

In contrast, when the information uncertainty is higher or the consequences are more unpredictable, users perceive a higher risk and become more vigilant. They scrutinize the source and content of health information more thoroughly. Zhang et al. (90) found that there was a significant positive correlation between the perceived riskiness of health information and the information avoidance behaviors during the COVID-19 pandemic. This indicates that users become more alert and cautious in the face of riskier information. At the same time, this motivates users to look for more evidence and support to distinguish the difference between misinformation, disinformation, rumors, and correct information. It helps avoid being misled by false or unreliable information. This vigilance helps users develop their discernment skills (91). Perceived value and perceived risk are relative concepts. Research showed that perceived costs and security risks have a great impact on customers’ perceived value (92). Therefore, the following assumptions are made:

H8: Perceived risk negatively influences perceived value.

H9: Perceived value positively influences health information discernment abilities.

H10: Perceived risk positively influences health information discernment abilities.

## 3 Materials and methods

### 3.1 Questionnaire design

This study designed a comprehensive questionnaire to investigate the factors influencing health information discernment abilities among users of online health communities. The questionnaire mainly consists of two parts: the basic demographic information of the respondent and the measurement scale. The demographic information part mainly involves gender, age, education level, living area, occupation, monthly income, health condition, attention to health information, and usage of OHCs. This study set up object screening items: excluding respondents who had never pay attention to health information. The measurement scale encompasses 13 variables: information quality, information sources, informational support, emotional support, technological security, technological facilitation, self-efficacy, perceived value, perceived risk, credibility discrimination ability, accuracy discrimination ability, reasonableness discrimination ability and support discrimination ability.

To ensure the reliability and validity of the questionnaire, the measures of the constructs were mainly adapted from well-established scales used in previous research. In addition, taking into consideration the characteristics of online health communities, the scenarios of questions were revised. The variables were measured using a 5-point Likert scale (1 = strongly disagree, 5 = strongly agree). Respondents are expected to provide answers based on their actual experiences and usage of online health communities. After completing the questionnaire preparation, four experts in the field were consulted and asked to review the narratives of the measurement items one by one and make corrections to any inappropriate statements, enhancing the scientific validity and rigor of the survey material. The final scale settings are shown in Table 1.

**Table 1:** Measurement items for the survey questionnaire.

Construct	Item labels	Items content	Source
Information quality	IQ1	The health information provided by OHCs is authentic and reliable.	Wang and Strong (116), Wixom and Todd (117)
	IQ2	The health information provided by OHCs is highly useful.	
	IQ3	The health information provided by OHCs is comprehensive.	
	IQ4	The health information provided by OHCs is easily comprehensible.	
Information source	ISE1	The health information provided by OHCs comes from credible sources.	Zhao and Du (118)
	ISE2	The health information provided by OHCs is based on scientific evidence.	
	ISE3	The health information provided by OHCs comes from knowledgeable sources.	
	ISE4	The health information provided by OHCs comes from reputable experts or authoritative institutions.	
Informational support	IST1	When I need to discern health information, the members of OHCs can provide me with advice.	Liang et al. (74)
	IST2	When I need to discern health information, members of OHCs can provide me with relevant evidence or clues.	
	IST3	When I need to discern health information, members in OHCs inform me about the causes.	
	IST4	When I need to discern health information, members of OHCs offer me correct information.	
Emotional support	ES1	When I need to discern health information, members of OHCs can provide me with encouragement and comfort.	Johnson and Lowe. (119)
	ES2	When I need to discern health information, members of OHCs will listen to my emotions.	
	ES3	When I need to discern health information, members of OHCs assist me, making me feel warm.	
	ES4	When I need to discern health information, members of OHCs help me alleviate my anxiety.	
Technological security	TS1	Using OHCs for health information discernment does not result in the exposure of user data.	Zhang et al. (120), Zhou (15)
	TS2	OHCs prioritize the utmost protection of user privacy for everyone.	
	TS3	Using OHCs for health information discernment brings me a great sense of reassurance.	
	TS4	OHCs possess the technical capabilities to ensure the security of information during the process of discernment.	
Technological facilitation	TF1	OHCs offer a wealth of functionalities and user-friendly interfaces that meet my needs for health information discernment.	Zlatolas et al. (121)
	TF2	OHCs offer multiple ways for discerning health information.	
	TF3	I prefer using feature-rich OHCs for health information discernment.	
	TF4	Specific modules in OHCs motivate me to actively discern health information.	
Self-efficacy	SE1	I am confident in addressing doubts about the accuracy of health information in OHCs.	Chen and Hung (122)
	SE2	I am confident in my ability to find reliable and trustworthy health information in OHCs.	
	SE3	I am confident to use correct health information from OHCs to make appropriate decisions regarding my health.	
	SE4	My knowledge and experiences are helpful in discerning health information in OHCs.	
Perceived value	PV1	Using OHCs for discerning health information has saved me time, energy, and money.	Yang et al. (123)
	PV2	Using OHCs for discerning health information brings me happiness.	
	PV3	Using OHCs for discerning health information has enhanced my knowledge of health and information.	
	PV4	Using OHCs for discerning health information has elevated my health literacy and information literacy.	
	PV5	Using OHCs for discerning health information has enhanced my ability to discern health information.	
Perceived risk	PR1	I am not worried about the health information on OHCs may mislead me.	Featherman and Pavlou (124)
	PR2	I am not worried about my physical health will be negatively affected.	
	PR3	I am not worried about experiencing a loss of energy, time, or financial resources.	
	PR4	I am not worried about others know about the disease I have and forming negative opinions about me.	
	PR5	I am not worried about my personal information being leaked or misused.	
Credibility discrimination ability	CDA1	When using OHCs, I check if the health information includes exaggerated or absolute statements.	Ivanitskaya et al. (125)
	CDA2	When using OHCs, I check if the health information includes any persuasive language.	
	CDA3	When using OHCs, I check if the health information includes promotional or advertising content.	
	CDA4	When using OHCs, I check if the health information includes statements claiming to provide unique or confidential information.	
	CDA5	When using OHCs, I check if the health information includes a lot of negations statements in health information.	
Accuracy discrimination ability	ADA1	When using OHCs, I check if the health information includes punctuation errors.	Harris (126)
	ADA2	When using OHCs, I check if the health information includes improper spacing.	
	ADA3	When using OHCs, I check if the health information includes grammar errors.	
	ADA4	When using OHCs, I check if the health information includes one-sided viewpoints.	
	ADA5	When using OHCs, I check if the health information is complete.	
Reasonableness discrimination ability	RDA1	When using OHCs, I check if the health information includes statements that intentionally overstate the importance.	Li et al. (127)
	RDA2	When using OHCs, I check if the health information includes a lot of emotional tone or verbal judgments.	
	RDA3	When using OHCs, I check if the logic of the health information makes sense.	
	RDA4	When using OHCs, I check if the content of the health information is relevant to its title or topic.	
Support discrimination ability	SDA1	When using OHCs, I check if the numerical statistics in the health information have an accurate source.	Zhang (25)
	SDA2	When using OHCs, I check if the health information includes the link of source documentation.	
	SDA3	When using OHCs, I check if the health information is false authority.	
	SDA4	When using OHCs, I pay attention to the timeliness of health information.	

### 3.2 Data collection

Before conducting the data survey, this study was approved by the Ethics Review Committee of the College of Life Sciences, Central South University (Reference no. 2023-1-34). This study mainly utilized the questionnaire website^1^ to conduct the survey. This study primarily conducted an online survey within China. According to the number of downloads of the applications in the App Store, we selected several representative and highly popular applications for distributing the questionnaire. For example, in China, WeChat had been downloaded 9.2 billion times; Sina Weibo had been downloaded 7.2 billion times, and QQ had been downloaded 6.1 billion times. These applications are all in Chinese language. They are not only served as social media platforms, but also a form of OHCs, facilitating quicker access to our target survey participants as many as possible. We employed a snowball sampling method to distribute the questionnaires by participating in topic discussions within WeChat/QQ health communication groups and subsequently inviting friends to share the questionnaires. Additionally, we initiated topic posts in Baidu Tieba forums such as Haodf Online Tieba and DXY Tieba, distributed the questionnaire through Sina Weibo by writing blog posts and commenting on others’ blogs, and utilized the comment functions on platforms like the WeDoctor WeChat official account and Meet You app to further disseminate the questionnaire.

Initially, a pre-survey was conducted with 113 users of online health communities to confirm the reliability and validity of the scale, which yielded favorable results and allowed for the formal survey to commence. The formal survey was conducted from September 7, 2023, to October 26, 2023. Finally, 772 questionnaires were received.74 respondents who had never paid attention to health information were excluded from the screening items. Furthermore, 43 questionnaires were removed due to the presence of abnormal data such as (1) choosing the same answer for all questions, (2) logical inconsistencies, (3) completing time <2 min, and (4) identical IP addresses for multiple respondents. This resulted in a final data set of 655 valid responses. According to the sample size requirements of the questionnaire survey, the sample size should be 5–10 times the number of scale question items (93), so the effective sample size of this study meets the requirements.

### 3.3 Analytical methods

This study primarily adopted the method of SEM for model validation. It is mainly used in social sciences, behavioral sciences, biostatistics, and other fields, aiding researchers deeply explore and understand potential causal relationships in complex data (94). Thus, SEM was used in this study to test hypotheses and analyze the research model. The data analysis involved two main steps. Firstly, the reliability and validity tests of the scale were conducted in this study. Reliability was evaluated using Cronbach’s alpha, calculated through SPSS, while convergent and discriminant validity were tested using Confirmatory Factor Analysis (CFA) via Amos 23.0. Secondly, the goodness of fit was calculated to analyze the effect size of the research model and the fit between the research model and the observed data. The magnitude and significance of the path coefficients were calculated to test the hypotheses of the theoretical model and to test the relationship among the latent variables (95).

SEM can identify and explore paths of influence between latent variables, examining both direct and indirect effects. It can better analyze the net effect of antecedent variables on the outcome variables. Inversely, it cannot analyze the complex causal relationships formed by the interdependence of multiple antecedent variables. In contrast, fsQCA assumes an asymmetric relationship between independent and dependent variables, acknowledging that multiple paths and solutions can lead to the same outcome. Based on SEM analysis, fsQCA supplements the exploration of how various influencing factors interact in a multiply and concurrently manner with the outcome variable (96). Studies have shown that the combination of SEM and fsQCA can provide a more comprehensive and in-depth analysis, enhancing the explanatory and predictive power of scientific theories (97). fsQCA is an analytical technique that combines fuzzy set theory with Boolean logic, which requires the data to be calibrated, and to be classified and degrees simultaneously through set affiliation. Then, the necessity and sufficiency analysis of the sample data is performed, from which finally the multifactorial combinations of the outcome variables are generalized to provide different theoretical paths for a given outcome (98).

## 4 Results

### 4.1 Sample characteristics

SPSS 25.0 was used to process the survey results. The demographic information of the survey respondents is shown in Table 2. The number of males is slightly higher than the number of females. 35.42% of the participants were aged between 18 and 40 years old. A total 65.50% of the participants had a bachelor’s degree or above. 74.40% are urban residents. The largest proportion of the participants were corporate workers, accounting for 34.66%. More than half of the participants had a monthly income of 10,000 RMB or lower. The physical health status of most participants is “General,” accounting for 34.96%. A total 40.00% of the participants indicated that they “Often” pay attention to health information. The highest proportion of participants (37.71%) reported using specialized online health websites, while 41.22% reported utilizing online health communities “5–6 times” per month.

**Table 2:** Demographic characteristics of participants.

Variables	Categories	Frequency (*N* = 655)	Percentage
Gender	Male	344	52.50%
	Female	311	47.50%
Age group	Below 18	65	9.93%
	18–40	232	35.42%
	41–65	199	30.38%
	66 and above	159	24.27%
Education level	Junior high school or below	96	14.65%
	Senior high school	130	19.85%
	Bachelor’s degree	326	49.77%
	Master’s degree or above	103	15.73%
Living area	Urban	487	74.40%
	Rural	168	25.60%
Occupation	Student	65	9.92%
	Government or State-owned enterprise employees	131	20.00%
	Private enterprise employees	227	34.66%
	Freelancer	30	4.58%
	Self-employed	65	9.92%
	Farming	26	3.97%
	Unemployed	29	4.43%
	Other	82	12.52%
Monthly income	Below 2000 RMB/month	95	14.50%
	2000–5000RMB/month	199	30.38%
	5,001–10000RMB/month	170	25.95%
	10,001–20000RMB/month	116	17.72%
	Above 20000RMB/month	75	11.45%
Health condition	Extremely poor	65	9.93%
	Relatively poor	87	13.28%
	General	229	34.96%
	Relatively good	164	25.04%
	Extremely good	110	16.79%
Attention to health information	Rarely	69	10.53%
	Sometimes	139	21.23%
	Often	262	40.00%
	Frequently	185	28.24%
Types of online health communities	Specialized online health websites (such as We Doctor, 39 Health, Haodaifu)	247	37.71%
	Health section of comprehensive websites (such as Health Section of Tencent Health and Bui Du)	144	21.98%
	Healthcare APP (such as DXY, Meet you)	161	24.58%
	Online health discussion groups (such as patient discussion QQ groups, WeChat groups)	103	15.73%
Frequency of using online health communities	1–2 times	66	10.08%
	3–4 times	135	20.61%
	5–6 times	270	41.22%
	7 or more times	184	28.09%

### 4.2 Structural equation modeling analysis

#### 4.2.1 Reliability and validity testing

The reliability of the scale was tested by SPSS25.0 of IBM, and the validity of the scale was analyzed by using Amos23.0. The results are shown in Tables 3, 4.

**Table 3:** Reliability and convergent validity testing.

Constructs	Items	Standardized loadings	Cronbach’s alpha	AVE	CR
Information quality(IQ)	IQ1	0.814	0.876	0.640	0.877
	IQ2	0.792			
	IQ3	0.799			
	IQ4	0.794			
Information source (ISE)	ISE1	0.774	0.869	0.625	0.870
	ISE2	0.787			
	ISE3	0.824			
	ISE4	0.777			
Informational support (IST)	IST1	0.804	0.878	0.643	0.878
	IST2	0.795			
	IST3	0.793			
	IST4	0.816			
Emotional support (ES)	ES1	0.809	0.884	0.655	0.884
	ES2	0.793			
	ES3	0.831			
	ES4	0.804			
Technological security (TS)	TS1	0.763	0.869	0.624	0.869
	TS2	0.799			
	TS3	0.791			
	TS4	0.805			
Technological facilitation (TF)	TF1	0.814	0.880	0.648	0.880
	TF2	0.820			
	TF3	0.792			
	TF4	0.793			
Self-efficacy (SE)	SE1	0.771	0.868	0.622	0.868
	SE2	0.783			
	SE3	0.784			
	SE4	0.817			
Perceived value (PV)	PV1	0.784	0.891	0.620	0.891
	PV2	0.785			
	PV3	0.807			
	PV4	0.785			
	PV5	0.776			
Perceived risk (PR)	PR1	0.839	0.914	0.680	0.914
	PR2	0.814			
	PR3	0.809			
	PR4	0.830			
	PR5	0.832			
Credibility discrimination ability (CDA)	CDA1	0.786	0.895	0.632	0.896
	CDA2	0.813			
	CDA3	0.786			
	CDA4	0.799			
	CDA5	0.793			
Accuracy discrimination ability (ADA)	ADA1	0.823	0.902	0.649	0.902
	ADA2	0.796			
	ADA3	0.793			
	ADA4	0.818			
	ADA5	0.798			
Reasonableness discrimination ability (RDA)	RDA1	0.818	0.881	0.651	0.882
	RDA2	0.799			
	RDA3	0.801			
	RDA4	0.809			
Support discrimination ability (SDA)	SDA1	0.792	0.882	0.652	0.882
	SDA2	0.807			
	SDA3	0.809			
	SDA4	0.821			

**Table 4:** Discriminant validity testing.

	IQ	ISE	IST	ES	TS	TF	SE	PV	PR	CDA	ADA	RDA	SDA
IQ	0.800												
ISE	0.376	0.791											
IST	0.400	0.333	0.802										
ES	0.312	0.332	0.341	0.809									
TS	0.375	0.348	0.380	0.365	0.790								
TF	0.380	0.313	0.371	0.312	0.330	0.805							
SE	0.428	0.351	0.324	0.369	0.359	0.359	0.789						
PV	0.380	0.355	0.362	0.365	0.431	0.347	0.384	0.787					
PR	−0.049	−0.167	−0.049	−0.069	−0.052	−0.045	−0.041	−0.040	0.825				
CDA	0.402	0.389	0.384	0.373	0.427	0.388	0.407	0.468	0.056	0.795			
ADA	0.404	0.391	0.386	0.375	0.429	0.390	0.409	0.470	0.056	0.610	0.806		
RDA	0.351	0.340	0.336	0.326	0.373	0.339	0.356	0.409	0.049	0.530	0.533	0.807	
SDA	0.392	0.379	0.375	0.364	0.417	0.379	0.397	0.457	0.054	0.592	0.595	0.518	0.807

It can be seen that the Cronbach’s Alpha varied from 0.869 to 0.914 for each construct (Table 3), exceeding the cut-off point of 0.70, demonstrating good scale reliability (99). The standardized loading values of all variables ranging from 0.771 to 0.839, exceeded the threshold of 0.6; the average variance extracted (AVE) of all latent variables was between 0.620 and 0.680, which is higher than 0.5; and the composite reliability (CR) values of all latent variables exceeded 0.7; confirming the satisfactory convergent validity of the scale (94). In Table 4, the values bold on the diagonal are the arithmetic square roots of the corresponding variables AVE. The values outside the diagonal indicate the correlation coefficients among the latent variables. According to the results, the arithmetic square root of AVE of all latent variables is higher than the correlation coefficients of the rows and columns in which they are located, which means that the discriminant validity among the latent variables is good (100).

#### 4.2.2 Model fit testing

The collected sample data were imported into AMOS software, and a model diagram was constructed to the analysis of model fit. The criteria for evaluating each model fit were referred to the recommendations of Dou et al. (101). The results are shown in Table 5. The ratio of chi-square to degrees of freedom (*X*^2^/DF) = 1.125, which is less than the recommended threshold of 3. The values of RMSEA, CFI, IFI, NFI, and GFI are 0.014, 0.992, 0.992, 0.930, and 0.920. All the fit indices are within the range of the recommended range, which indicates that the research model fits well with the collected data.

**Table 5:** Goodness-of-fit results.

Fit indices	*X*^2^/DF	RMSEA	CFI	IFI	NFI	GFI
Recommended values	<3	<0.08	>0.9	>0.9	>0.9	>0.9
Model values	1.125	0.014	0.992	0.992	0.930	0.920

#### 4.2.3 Hypothesis testing

The critical ratio (CR) value is the ratio of the parameter estimate to the parameter estimate standard error (SE). If the CR > 1.96, then the parameter estimate passes the test at the significance level *α* = 0.05.

**Figure 2:**
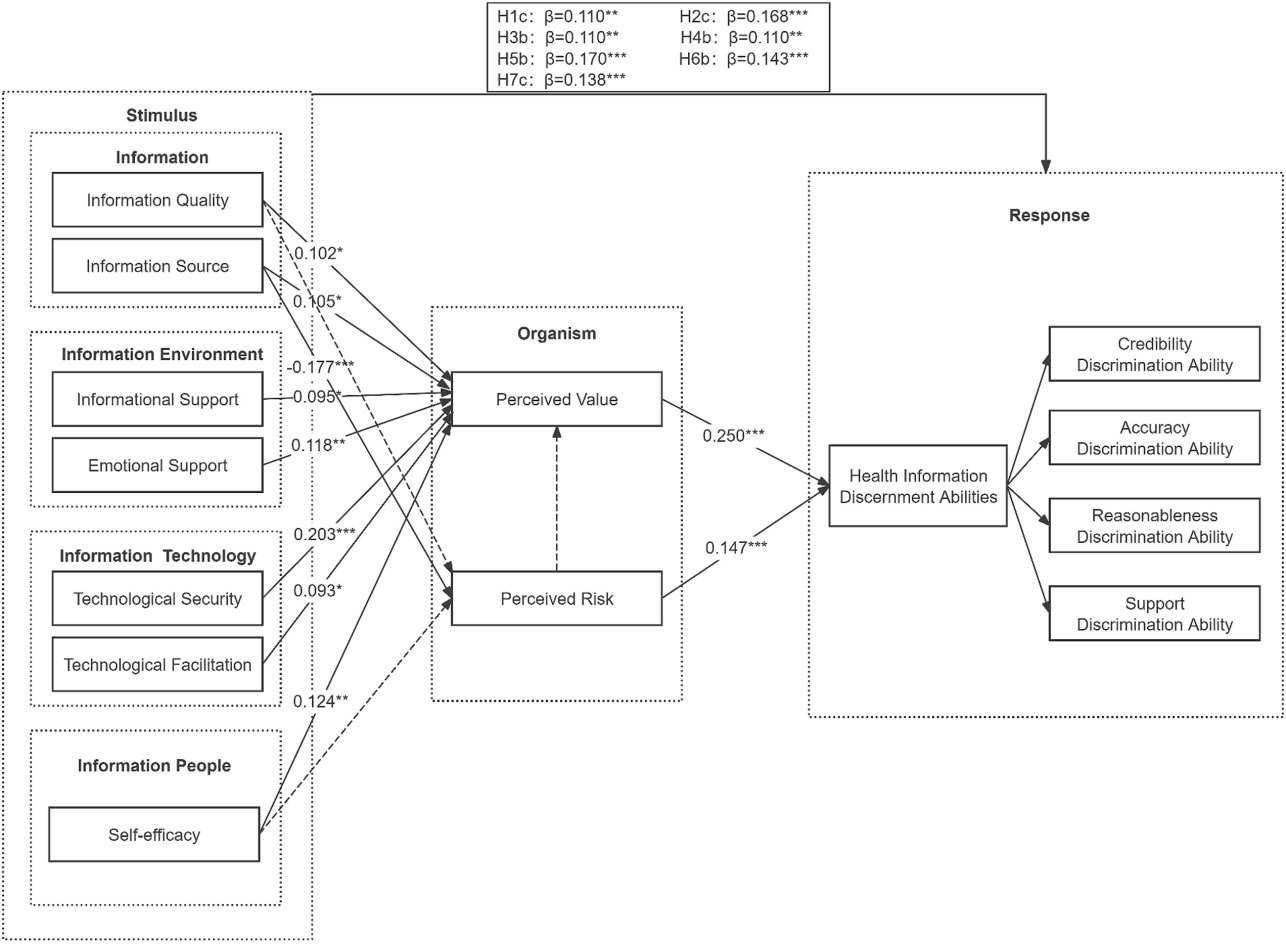
Research model of hypothesis testing. Solid arrows indicate having a significant impact, while dashed arrows represent the absence of an impact.

According to the C.R value and *p*-value shown in Table 6, among the 20 hypotheses, 17 hypotheses were found to be significant at *α* = 0.05 level. While the remaining three hypotheses did not pass the test. The specific test results are as follows: The information source has a significant negative effect on perceived risk. The information quality, information source, informational support, emotional support, technological security, technological facilitation, and self-efficacy have a significant positive effect on perceived value. Information quality, information source, informational support, emotional support, technological security, technological facilitation, and self-efficacy have a significant positive effect on health information discernment abilities. Perceived value and perceived risk have a significant positive effect on health information discernment abilities. However, the hypothesis of the effect of information quality on perceived risk did not pass the test. The influence of self-efficacy on perceived risk is not significant. The influence of perceived risk on perceived value is also not significant (Figure 2).

**Table 6:** Hypothesis testing results of the research model.

Hypothesis	Path	Standardized coefficient	S.E.	C.R.	*P*	Result
H1a	IQ → PV	0.102	0.046	2.117	*	Support
H1b	IQ → PR	0.011	0.054	0.205	0.838	Not support
H1c	IQ → HIDA	0.110	0.034	2.582	**	Support
H2a	ISE → PV	0.105	0.046	2.278	*	Support
H2b	ISE → PR	−0.177	0.055	−3.556	***	Support
H2c	ISE → HIDA	0.168	0.035	4.061	***	Support
H3a	IST → PV	0.095	0.044	2.042	*	Support
H3b	IST → HIDA	0.110	0.033	2.683	**	Support
H4a	ES → PV	0.118	0.042	2.627	**	Support
H4b	ES → HIDA	0.110	0.031	2.763	**	Support
H5a	TS → PV	0.203	0.043	4.304	***	Support
H5b	TS → HIDA	0.170	0.033	3.995	***	Support
H6a	TF → PV	0.093	0.042	2.053	*	Support
H6b	TF → HIDA	0.143	0.031	3.542	***	Support
H7a	SE → PV	0.124	0.043	2.620	**	Support
H7b	SE → PR	0.017	0.052	0.329	0.742	Not support
H7c	SE → HIDA	0.138	0.033	3.256	***	Support
H8	PR → PV	0.013	0.034	0.334	0.738	Not support
H9	PV → HIDA	0.250	0.036	5.780	***	Support
H10	PR → HIDA	0.147	0.026	4.299	***	Support

*p < 0.05, **p < 0.01, ***p < 0.001.

### 4.3 Fuzzy-set qualitative comparative analysis

#### 4.3.1 Data calibration

To categorize the sample data into different set affiliations, a data calibration operation must be performed on the questionnaire data before the configuration analysis. In this study, a 5-point Likert scale was chosen for the design of the measurement questionnaire, which had to be transformed into an affiliation scale between 0 and 1 (102). By calculating the mean value of the measurement items as the original data of each variable. fsQCA4.1 software was used to calibrate the integrated data, with anchor points set at (0.95, 0.5, 0.05), which means that 95% of the set is fully affiliated, 50% is the maximum fuzzy affiliation point, and 5% is not affiliated at all (103).

#### 4.3.2 Necessary analysis of single conditions

A single condition necessity test was carried out on the outcome variables, so as to explore whether there is a single necessary condition variable that leads to the outcome. It is generally considered that consistency >0.9 can be regarded as a necessary condition. The results are shown in Table 7. It can be seen that the consistency of Information Quality (IQ), Information Source (ISE), informational support (IST), Emotional Support (ES), Technological Security (TS), Technological Facilitation (TF), Self-Efficacy (SE), Perceived Value (PV), and Perceived Risk (PR) was <0.9, which indicates that the explanatory power of these nine single elements on the outcome variables is weak. They did not have a dominant influence on health information discernment abilities. Further exploration is needed to examine the sufficiency of the effects of configurations on the outcome.

**Table 7:** Results of single conditions necessity testing.

Antecedent variables	Outcome variable	
	HIDA	
	Consistency	Coverage
IQ	0.790	0.822
~IQ	0.506	0.746
ISE	0.788	0.811
~ISE	0.504	0.755
IST	0.787	0.812
~IST	0.507	0.756
ES	0.780	0.814
~ES	0.505	0.741
TS	0.782	0.832
~TS	0.521	0.745
TF	0.780	0.821
~TF	0.512	0.742
SE	0.790	0.822
~SE	0.504	0.742
PV	0.829	0.827
~PV	0.474	0.744
PR	0.626	0.797
~PR	0.659	0.771

#### 4.3.3 Sufficiency analysis of configurations

The truth table was constructed based on the calibrated fuzzy set data table. The consistency threshold is set to 0.80. The case threshold is set to 3. The PRI consistency threshold is set to 0.75 (104). Typically, three kinds of solutions are generated after analyzing through fsQCA4.1: complex solutions, intermediate solutions, and parsimonious solutions. The parsimonious solutions and the intermediate solutions can effectively balance the discrepancy between theory and facts (105). Thus, this study combined the parsimonious solutions and the intermediate solutions as the analysis results. The condition variables are distinguished into core conditions and peripheral conditions through qualitative comparative analysis. There are 10 specific condition variable combination paths, which are divided into four types, as shown in Table 8.

**Table 8:** Results of configuration analysis.

Antecedent Variables	S1(ISE*IST)		S2(TS*TF)		S3(ISE*IST*TS*TF)				S4(ISE* ~ TF*PR)	
	S1a	S1b	S2a	S2b	S3a	S3b	S3c	S3d	S4a	S4b
IQ	•	•	•	•	•	•		•	⊗	⊗
ISE	●	●		•	●	●	●	●	●	●
IST	●	●	•		●	●	●	●	●	⊗
ES	•	•	•	•	•		•	•	⊗	•
TS	•		●	●	●	●	●	●	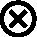	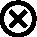
TF		•	●	●	●	●	●	●	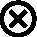	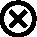
SE	•	•	•	•		•	•	•	⊗	⊗
PV	•	•	•	•	•	•	•		•	•
PR		⊗		⊗		⊗	⊗	●	●	●
Raw coverage	0.441	0.342	0.439	0.343	0.433	0.345	0.342	0.314	0.182	0.188
Unique coverage	0.022	0.011	0.024	0.011	0.014	0.014	0.009	0.008	0.008	0.011
Consistency	0.991	0.995	0.992	0.995	0.994	0.993	0.992	0.995	0.993	0.991
Solution consistency	0.976									
Solution coverage	0.574									

“●” indicates that the core conditions exist. “
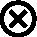
” indicates that the core conditions absent. “•” indicates that the peripheral conditions exist. “⊗” indicates that the peripheral conditions absent. Blank space indicates that the conditions can exist or not.

According to Table 8, the consistency of the configurations ranges from 0.991 to 0.995, and the solution consistency level of the health information discernment abilities is 0.976, with a solution coverage of 0.574. This indicates that the collection of these 10 paths has a strong explanatory power of the health information discernment abilities. It is able to explain 57.4% of the results of the sample. By categorizing configurations with the same core conditions, four types of configuration patterns for health information discernment can be identified as follows:

1. Configuration S1: This configuration consists of two sub-configurations (S1a, S1b). The common core conditions are high information sources and high informational support. Common peripheral conditions are high information quality, high emotional support, high self-efficacy, and high perceived value. In addition to these shared core and peripheral conditions, when there is high technological security, or high technological facilitation and low perceived risk coexist, both situations can trigger the generation of high health information discernment abilities.
2. Configuration S2: This configuration consists of two sub-configurations (S2a, S2b).The common core conditions are high technological security and technological facilitation. Common peripheral conditions are high information quality, high emotional support, high self-efficacy, and high perceived value. In addition to these shared core and peripheral conditions, when there is high informational support, or high information sources and low perceived risks coexist, both situations can trigger the generation of high health information discernment abilities.
3. Configuration S3: This configuration consists of four sub-configurations (S3a, S3b, S3c, S3d). The shared core conditions are high information source, high informational support, high technological security, and high technological facilitation. Comparing S3a and S3d, we found that the shared peripheral conditions are high information quality and high emotional support. When there is a high perceived value or the coexistence of high self-efficacy and high perceived risk, both situations can trigger high health information discernment abilities. Comparing S3b and S3c, we found that the shared peripheral conditions are high self-efficacy, high perceived value, and low perceived risk. When both the shared core and peripheral conditions are present, high information quality or high emotional support triggers high health information discernment abilities, suggesting a substitutable relationship between information quality and emotional support.
4. Configuration S4: This configuration includes two sub-configurations (S4a, S4b). The shared core conditions are high information source, low technological security, low technological facilitation, and high perceived risk. The shared peripheral conditions are low information quality, low self-efficacy, and high perceived value. Comparing S4a and S4b, we found that when both the shared core and peripheral conditions are present, high informational support or high emotional support triggers high health information discernment abilities, indicating a substitutable relationship between informational support and emotional support.

In summary, it can be seen from the 10 sub-configurations that while nine antecedent conditions all appear in the same configuration at the same time. These configurations contain at least two influencing factors. It shows that the health information discernment abilities of OHCs users are not caused by a single influencing factor but rather by the interdependence of multiple factors. There are multiple configurations that can lead to the target results. Traditional SEM studies cannot explain the complex relationship between these factors and outcomes.

#### 4.3.4 Robustness testing

The robustness testing is necessary to ensure that the findings are generalizable. Schneider and Wagemann (106) proposed feasible ways of robustness testing, such as by adjusting the data calibration thresholds and the consistency thresholds, and by changing the frequency of cases. In this study, the consistency threshold was changed from 0.8 to 0.85. It was found that the parameter adjustments did not result in any changes in the configuration paths as well as in the fit parameters for consistency and coverage. Thus, the analysis outcomes was found to be relatively robust.

## 5 Discussion

### 5.1 Main findings

1. The four dimensional factors of information (information quality, information source), information environment (informational support, emotional support), information technology (technological security, technological facilitation), and information people (self-efficacy) all have significant positive effects on health information discernment abilities. As with previous studies, online health communities are interlocking information systems. The information activities of users cannot be separated from the influences of all four factors: health information, community environment, information technology, and community users (107). From the viewpoint of information factors, high-quality information resources save users the energy and time needed for information screening, and authoritative information sources are more convincing to users (108). In terms of the information environment, the strong informational support of the community platform facilitates the efficient delivery of online health information and increases users’ health knowledge, while sufficient emotional support helps users to relieve anxiety and get inspired. From the viewpoint of information technology, information technology links users with the community environment. The assistance of advanced information technology makes the community function perfectly and enriches the user experience, thus improving the efficiency of users in identifying health information. Technological security also guarantees that the privacy of users is protected (109). From the viewpoint of the information people, the user is the crucial element in the entire health information discernment process. The user’s health knowledge reserve, information searching skills, and information evaluation ability all reflect the user’s self-efficacy (110). When the user’s self-efficacy is higher, it is easier to obtain true and effective health information. Analyzing from the perspective that information ecological elements such as information, information environment, information technology, and information people fit together can more comprehensively and deeply analyze the influencing factors of health information discernment abilities.
2. Perceived value and perceived risk positively influence users’ health information discernment abilities. However, perceived risk does not influence perceived value. The path coefficients of perceived value and perceived risk on health information discernment abilities are 0.250 and 0.147, respectively, with *p* < 0.001. This indicates that perceived value and perceived risk have a significant influence on health information discernment abilities, with perceived value having a much greater effect than perceived risk. On the one hand, when users engage in health information discernment, they weigh the trade-off between value and risk. Higher value perception encourages users to seek out health information, while higher risk perception leads them to avoid it (111). In the process of judging the value and risk of health information, users’ health literacy improves continuously, leading to enhanced health-related knowledge and cognitive skills, which in turn bolster their discernment abilities. On the other hand, it is believed that users’ primary motivation for discerning health information is to obtain information that aligns with their personal needs and health goals (112). Compared with perceived risk, users place more importance on the value they can obtain after discerning health information. Thus, perceived value plays a greater role, weakening the influence of perceived risk.
3. The effect of information quality on perceived risk is not significant. However, information quality has a significant positive influence on perceived value and health information discernment abilities. Consistent with the results of fsQCA analyses, in configuration S3d, high information quality promotes high health information discernment abilities when high information quality is used as a peripheral condition and high perceived risk is used as a core condition. In configurations S1, S2, S3a, and S3b, high information quality and high perceived value, as both peripheral conditions, can lead to the generation of high health information discernment abilities. This May be because most of the types of health communities used by the users in this study were from professional online health websites, such as Seeking Medicine, We Doctor, and Haodaifu. For users, compared to other online health information exchange zones or groups, such as health discussions on Sina Weibo, health information obtained through professional health websites is more medically authoritative and instructive (113). Users have sufficient trust in professional health websites, thus it will not have an impact on their risk perception. However, it will be convenient for users and increase their perceived value.
4. Self-efficacy does not have a significant effect on perceived risk, but it does have a significant positive effect on perceived value and health information discernment abilities. In configuration S3d, high self-efficacy as a peripheral condition and high perceived risk as a core condition can lead to high health information discernment abilities. In configurations S1, S2, S3b, and S3c, high self-efficacy and high perceived value were both used as peripheral conditions that could lead to high health information discernment abilities. The results of the survey showed that 65.50% of the participants had a bachelor’s degree or above. The higher the education level of the users, the more health information discernment skills they master, which encourages they to quickly obtain high-value information. When users have acquired a sufficient amount of health knowledge through reliable sources and are self-assured in their understanding, they are better equipped to assess and navigate risky health information effectively (114). They rely on their solid foundation of health knowledge to critically evaluate the provided information, enabling them to avoid falling victim to any potential health risks or misinformation. Their confidence empowers them to make informed Decisions about their wellbeing, carefully considering the credibility and validity of the health information they encounter, and subsequently taking appropriate actions to safeguard their health and avoid potential harm (115). In terms of age distribution, the 18–40-years-old group has the largest proportion, and they are full of hope for the future, positive and optimistic, and usually have a lower propensity to perceive risk.
5. There are 10 antecedent configurations affecting users’ ability to screen health information, categorized into 4 types. The core conditions of configuration S1 are high information source and high informational support. For configuration S2, the core conditions are high technological security and high technological facilitation. The core conditions of configuration S3 are high information source, high informational support, high technological security, and high technological facilitation. The core conditions of configuration S4 are high information source, low technological facilitation, and high perceived risk. The four types of configurations illustrate that information source, informational support, technological security, technological facilitation, and perceived risk are the core conditions affecting users’ health information discernment abilities. Additionally, information quality, emotional support, self-efficacy, and perceived value appeared several times as peripheral conditions; further supporting the fact that they significantly affect users’ health information discernment abilities.

In addition, the consistency and coverage of S1, S2, and S3 were higher than that of S4. It is because the components of S1, S2, S3b, S3c, and S3d all contained the factors of information, information environment, information technology, and information people. While the component of S3a contained the factors of information, information environment, and information technology. In contrast, configuration S4 only includes information and information environment factors. This further confirms the applicability of the information ecology theory in online health communities, where the triggering of high health information discernment abilities requires a combination of information factors such as information, information environment, information technology, and information people, rather than a single factor.

Although perceived value has the greatest effect on health information discernment abilities in the SEM single-factor analysis. It is a peripheral condition in all four configurations, which suggests that it does not play a significant role when combined with other factors. This finding compensates for the limitations of SEM in examining the net benefit of a single factor.

### 5.2 Theoretical significance and practical implications

Theoretically, this study applied the stimulus-organism-response theory, information ecosystem theory, and the Mindsponge theory to the field of health information discernment abilities research, providing rich and solid theoretical support for health information discernment abilities research. The study considered the online health community as an information ecosystem, which extends the research perspective on health information discernment abilities. In addition, this study attempted to apply a combination of questionnaire survey and fsQCA methods to the study of health information discernment abilities. Previous studies have mainly used the experimental method or interview method, and seldom used the fsQCA method. This study expanded the applicability of the fsQCA method and enriched the research method system of users’ health information discernment abilities.

Practically, it provides a reference for improving users’ health information discernment abilities. From the information perspective, it is necessary to strengthen the construction of information resources in online health information communities, raise the threshold of information release, and set community norms to constrain users to ensure the circulation of high-quality information resources and cut off the sources of poor-quality health information. At the same time, it is necessary to encourage more professional doctors, medical researchers, and health experts to join the community to provide users with accurate health information. From an information environment perspective, attention should be paid to users’ needs and understanding of users’ interests and preferences, so as to provide personalized services and enhance user engagement. Establishing channels for friendship and mutual assistance, so that users can communicate freely in the community, fostering strong friendships among users, thus increasing their sense of belonging and affiliation to the community. From an information technology perspective, it is necessary to increase the strength of information review and information regulation to detect and deal with false or harmful information on time. Online health communities should provide users with information screening tools and reliable information resources, such as guidance on assessing the credibility of websites and recommending authoritative health websites and applications. From a user perspective, users’ health information literacy education should be strengthened. Training and education courses on health information discrimination should be provided to users to improve their sense of self-efficacy.

## 6 Conclusion

This study explored the factors influencing health information discernment abilities and the configuration paths. From the results of the SEM analysis, it was found that information quality, information source, informational support, emotional support, technological security, technological facilitation, self-efficacy, perceived value, and perceived risk all have a positive effect on health information discernment abilities. Improvement in health information discernment abilities requires a combination of factors, not just a single element. The 10 paths analyzed by fsQCA contain a minimum of two dimensional factors in each combination of information, information environment, information technology, and information people. fsQCA supplements the exploration of relationships between multiple variables, complementing the SEM approach. The combined application of the two methods should be promoted. This study hopes to provide experiences and references for online health community information service, information resource construction, and users’ health information discernment abilities development.

## 7 Limitations

The primary data of this study were mainly derived from people using online health communities in China, which lacked an exploration of multiculturalism. User information behaviors and cultural practices May be different in other countries or regions. Future studies May consider sending questionnaire emails to users in different countries to obtain data. In addition, it is difficult to avoid subjective bias and the influence of the environment in which the questionnaires are completed. In future studies, empirical analyses could be conducted in conjunction with data crawled from online health communities or user interviews. The primary demographic for this survey was the youth, with an uneven distribution across other age groups. However, as the influence of online health communities continues to grow, people of different age groups are increasingly paying attention to these platforms. In the future, with sufficient time and resources, the sample survey range can be expanded to study the health information discernment abilities of users across different demographic groups.

## Data Availability

The raw data supporting the conclusions of this article will be made available by the authors, without undue reservation.

## Footnotes

^1^ https://www.wenjuan.com/s/IzaM3e4
